# Epidemiology of Irritable Bowel Syndrome in a Large Academic Safety-Net Hospital

**DOI:** 10.3390/jcm13051314

**Published:** 2024-02-26

**Authors:** Kathleen Cheng, Christina Lee, Ramune Garniene, Howard Cabral, Horst Christian Weber

**Affiliations:** 1 Department of Medicine, Boston Medical Center, Boston, MA 02118, USA; 2 Department of Medicine, Beth Israel Deaconess Medical Center, Boston, MA 02118, USA; 3 Middleboro Pediatrics, Lakeville, MA 02347, USA; 4 Department of Biostatistics, Boston University School of Public Health, Boston, MA 02118, USA; 5 Section of Gastroenterology, Chobanian & Avedisian School of Medicine, Boston University, Boston, MA 02218, USA

**Keywords:** irritable bowel syndrome, epidemiology, racial disparities, safety-net hospital

## Abstract

(1) Background: Irritable bowel syndrome (IBS) is a highly prevalent disorder of gut–brain interaction (DGBI) that is known to reduce the quality of life and raise healthcare costs. The aim of this study was to describe the epidemiology of IBS in a large multiracial academic safety-net hospital. (2) Methods: An electronic query was performed using ICD-9 codes to identify 740 IBS outpatients seen at the Boston Medical Center (BMC) between 1 January 2005 and 30 September 2007. Demographic data were collected from electronic medical records. Bivariate analyses using chi-square tests and ANOVA were used to calculate the significance of categorical and continuous dependent variables, respectively. (3) Results: Compared with the general BMC outpatient population, the IBS cohort consisted of significantly higher proportions of White and Asian patients and lower proportions of Black and Hispanic patients (*p* < 0.0001). White and Asian patients predominantly had private insurance, while Black and Hispanic patients mostly had government/state-funded or no insurance (*p* < 0.0001). The IBS subgroup frequencies were similar across racial groups; however, Hispanic patients had IBS with constipation (32%, *p* < 0.02) more often compared to non-Hispanic patients. (4) Conclusions: Significant differences were found across the racial groups studied in this large outpatient IBS cohort. These findings are likely attributed to racial and socioeconomic disparities in healthcare access and utilization.

## 1. Introduction

Irritable bowel syndrome (IBS) is a disorder of gut-brain interaction (DGBI) characterized by abdominal pain with altered bowel habits in the absence of structural and biochemical abnormalities [1]. It is a chronic relapsing condition that affects up to 11% of the global population and 16.7–24.2% of the United States population [2,3]. The Rome criteria, a symptom-based criteria first established in the late 1980s, continues to guide the diagnosis of IBS [4,5]. Per the most recent iteration of these clinically based criteria, the Rome IV criteria, IBS subtypes are classified by predominant bowel patterns and include IBS with diarrhea (IBS-D), IBS with constipation (IBS-C), IBS with mixed bowel habits (IBS-Ms), and unsubtyped IBS (IBS-U) [5,6].

IBS is a high-burden disease associated with several intra- and extra-intestinal comorbidities [7,8,9]. It is strongly associated with functional impairment and mood, anxiety, and sleep disorders [10]. Patients with IBS have been found to have health-related quality of life reductions comparable to those associated with gastroesophageal reflux disease, diabetes, and end-stage renal disease [11]. They are also more likely to undergo invasive procedures and surgeries, with three-fold rates of cholecystectomy and two-fold rates of appendectomy and hysterectomy compared to the general population [12]. Overall, patients with IBS incur about 50% more direct healthcare costs compared with non-IBS patients, leading to direct and indirect costs exceeding 20 billion USD in the United States from 1994 to 1995 [13,14]. Despite the severe illness burden on both the individual IBS patient and the health care system at large, research characterizing the epidemiology of IBS in the US population has remained minimal, specifically concerning different racial groups.

Previous studies found high variability in IBS prevalence rates across different geographic locations. In a prior meta-analysis, prevalence rates ranged from 21.0% in South America to 15.0% in South Europe, 11.8% in North America, and 7.0% and 7.5% in Southeast Asia and the Middle East, respectively [3]. These geographical variations were attributed to regional differences in diet, access to care, and cultural awareness of IBS [2]. Interestingly, cross-sectional studies in Thailand and Singapore found increasing IBS prevalence rates over the past decades, a trend that has been attributed to increasing global adaptation to Westernized lifestyles and diets [15,16,17]. Study findings have also varied with the use of different diagnostic criteria and surveying practices [18].

The majority of epidemiological studies were conducted in Northern Europe and focused primarily on monoracial White populations, thus lacking generalizability to multiracial populations. As a result, racial or ethnic variations in IBS prevalence rates have yet to be identified. Community studies conducted in Singapore and Malaysia found no significant differences in prevalence rates of IBS among participants of Chinese, Malaysian, and Indian ethnicity [16,19]. Existing data on IBS prevalence rates across different racial groups in the United States are very limited and contradictory. In a questionnaire-based study in 1995, no significant differences in prevalence rates were found between White and Black college students in Alabama [20]. Another study in 2005 found higher prevalence rates of IBS in White compared to Black adults in Jackson, Mississippi [21]. However, the opposite finding was reported in a 2007 study involving elderly residents in the Chicago Health and Aging Project [22]. The prevalence of IBS among Hispanic and Asian populations within the US is still unknown, although a survey-based study in 1993 reported fewer irritable bowel-type symptoms among Asian participants compared to other ethnic groups [23].

The symptom profile of IBS has also been found to vary significantly across different geographic locations. A 2008 study involving eight countries found that Mexican, Chinese, and Italian participants experienced constipation, diarrhea, and bloating as their predominant IBS symptoms, respectively [24]. Such differences are likely culturally significant as they have been found to persist when comparing different ethnic populations in the same country: for example, constipation and diarrhea-dominant bowel pattern subtypes were found to be more prevalent in White Americans compared to Asian American women [25]. Although a systematic review in 2005 reported an even distribution of IBS-C, IBS-D, and IBS-M subtypes among IBS patients in the US, there has not yet been a comprehensive study describing the distribution of IBS subtypes across different racial groups [26].

IBS is a highly prevalent condition that impacts the quality of life and healthcare expenditures globally [15]. While the pathophysiology of IBS is complex and multifactorial, it is known to be influenced by many racial and cultural factors, including genetics, diet, and gut microbiome composition [27]. Because prior epidemiological studies have largely been limited to single-race populations, the impact of racial patterns in IBS remains poorly understood. The aim of our study was to describe the epidemiology of IBS and its subtypes in a large multiracial academic safety-net hospital in the United States. Our secondary aim was to examine various demographic characteristics, including age, BMI, sex, and insurance status of IBS patients across different racial and ethnic groups.

## 2. Materials and Methods

### 2.1. Study Design

A cross-sectional, population-based retrospective study was conducted at the Boston Medical Center (BMC), an urban academic medical center that serves as the largest safety-net hospital in New England. This study was approved by the BMC Institutional Review Board and was in accordance with the ethical standards of the institutional and national research committee and with the 1995 Helsinki Declaration and its later amendments or comparable ethical standards. Due to the retrospective nature of the study and the preserved anonymity of patients, a waiver of informed consent was obtained from and approved by the BMC IRB.

Seven hundred and forty patients with IBS between 1 January 2005 and 30 September 2007 were identified by performing an electronic query for ICD-9 code diagnosis of irritable bowel syndrome (564.1) in patients aged 18 years and older. Patients under the age of 18 were excluded from the study. An electronic chart review was performed to further subcategorize these patients using a four-subtype classification system (IBS-C, IBS-D, IBS-M, and IBS-U) based on documented symptoms and the Rome III criteria, the most updated IBS clinical criteria during the study period [4]. Demographic data, including age, BMI, sex, and insurance status, were obtained from BMC’s electronic medical record.

### 2.2. Statistical Methods

In order to examine the univariate distributions of the data, we generated descriptive statistics using counts and percentages for categorical variables and means, standard deviations, and minima/maxima for continuous variables. We next performed bivariate analyses using chi-square tests of significance for cross-tabulations of categorical variables, e.g., sex, marital status by race/ethnicity group, and one-way analyses-of-variance (ANOVA) with post hoc comparisons following statistically significant global tests via Tukey’s studentized range procedure for continuous dependent variables by categorical variables, e.g., mean age and BMI by race/ethnicity group. All analyses were performed using SAS for Windows, version 9.2 (ref) 2008 SAS Institute, Inc., Cary, NC, USA. The *p*-values less than 0.05 were deemed statistically significant.

## 3. Results

Of the 740 patients enrolled in the study, 492 were White (70%), 99 were Black (14%), 44 were Asian (6%), and 65 were Hispanic (9%). Forty patients did not indicate their race. When compared with the racial distribution of the BMC outpatient population in 2007, the IBS cohort consisted of significantly higher proportions of White and Asian patients and lower proportions of Black and Hispanic patients (*p* < 0.0001) (Table 1). These differences remained significant when compared with the racial distribution of the annual BMC unique outpatient population in 2005 and 2006, which remained consistent with that of 2007 (*p* < 0.0001) (Table 2).

The demographic characteristics of the IBS cohort are summarized in Table 3. The mean age [±SD] of all IBS patients was 43 [±15] years, with almost three-quarters of the study cohort being age 30 or older. The average age at the time of diagnosis was 37 [±14] years. Black and Hispanic patients were diagnosed with IBS at a later age (40 [±17] years and 42 [±15] years, respectively) compared to White and Asian patients (36 [±14] years and 37 [±14] years), which was not statistically significant (*p* = 0.06).

The mean BMI [±SD] of the entire IBS cohort was 27 [±7] kg/m^2^. The average BMI of Black patients with IBS was significantly higher at 30 [±8] kg/m^2^ compared to that of White (27 ± 7), Hispanic (27 ± 5), and Asian (23 ± 4) IBS patients (*p* < 0.0001). Conversely, the average BMI of Asian patients with IBS was significantly lower than that of other racial groups (Table 3; *p* < 0.0001). When stratified by BMI-based classification, 43% of Black IBS patients were in the obese category, while 64% of Asian IBS patients were in the normal category (Table 4) [28].

Seventy-five percent of all IBS patients were female without significant differences across racial groups. The majority of Hispanic and Asian patients with IBS were married (43% and 52%, respectively) while the majority of White and Black patients with IBS were single (58% and 59%, respectively). Black and Hispanic IBS patients were more likely to have children than White and Asian patients. There was no significant difference in smoking history or employment status across all races in the IBS cohort.

Eighty-six percent of all IBS patients had either private insurance or Medicaid/Medicare. White and Asian patients with IBS predominantly had private insurance, whereas more than half of Black (54%) and Hispanic (63%) patients had Medicaid/Medicare or no insurance (*p* < 0.0001). Data on the education levels were missing for most patients in the IBS cohort (541 patients). Among those with recorded data, over 50% of White and Asian patients had graduate/professional education while over 50% of Black and Hispanic patients had college or graduate/professional education.

IBS subtype frequencies were similar across the four racial groups (Table 5). However, Hispanic patients had a higher frequency of IBS-C when compared with all non-Hispanic IBS patients (*p* < 0.02) (Table 6). Of the 44 Hispanic patients with IBS without constipation, 11 (25%) had IBS-D, 11 (25%) had IBS-M, and 22 (50%) had IBS-U. Of the 507 non-Hispanic patients with IBS without constipation, 167 (33%) had IBS-D, 80 (16%) had IBS-M, and 260 (51%) had IBS-U.

## 4. Discussion

To the best of our knowledge, this is the first study to describe the in-depth epidemiology of IBS in a large multiracial population in the United States. Compared to the general unique outpatient population of the Boston Medical Center, our large cohort of 740 IBS patients had significantly higher proportions of White and Asian patients and lower proportions of Black and Hispanic patients. While prior data on racial characteristics of IBS are limited and mixed, findings from the current study affirm two previous studies from 1990 and 2005 which found IBS to be 5.3 and 2.5 times more prevalent in White adults compared to Black adults, respectively [21,29]. One of the critical strengths of our study is the inclusion of all major racial/ethnic groups permitting direct comparison of epidemiological and demographic characteristics as reported here, given that there was no prior existing data comparing IBS features among multiple racial groups.

Previous studies found the highest rates of IBS in those aged 45–64 [29]. Similarly, the mean age of our cohort at the time of enrollment was 43 years, suggesting IBS to be more prevalent among older adults. However, fifty percent of IBS patients are reported to experience onset of symptoms before the age of 35 years, suggesting that diagnosis of IBS is often delayed [2].

Though obesity has been associated with an increased risk of IBS, a 2014 review article found prevalence rates of IBS in obese patients to vary depending on the population being studied [30,31,32]. This finding was affirmed in our study, which observed higher average BMI in Black IBS patients and lower average BMI in Asian IBS patients. Compared to the general US population in 2003–2004, our IBS cohort had lower prevalence rates of overweight or obesity in White participants and similar prevalence rates in Black participants [33]. However, without data on obesity rates of the general outpatient population to use for comparison, the clinical significance of this finding remains uncertain.

Previous studies have consistently found IBS to be more prevalent in women than men, with the only exception being in India and Sri Lanka, where IBS was diagnosed more frequently in men than in women [34,35]. Our study is also the first to determine similarly observed higher proportions of female patients for IBS patients across all studied racial/ethnic groups.

In our study, Asian and White patients, who were overrepresented in this IBS sample, predominantly had private insurance, whereas Hispanic and Black patients, who were underrepresented as compared with the general unique outpatient population in this large safety-net hospital, predominantly had government/state-funded healthcare insurance or no insurance. Insurance payer status is a known and reliable marker of socioeconomic status (SES) and healthcare access and was available for all but one of the patients in our study cohort [36,37]. Black and Hispanic IBS patients also tended to have lower education levels than White and Asian IBS patients, which may have been confounded by the fact that college students of the affiliated university were included in this sample. Overall, IBS prevalence rates were found to be significantly lower among groups with indicators of lower socioeconomic status.

Prior data on the relationship between IBS and SES are mixed. A 1998 study identified privileged childhood living conditions as a risk factor for the development of IBS. However, in a subsequent study in 2005, lower income and education levels were directly correlated with IBS prevalence rates [21]. Lower SES is widely associated with chronic stress, which in turn has been implicated in the development and exacerbation of visceral pain disorders such as IBS [38,39,40]. IBS patients with lower SES have been shown to have higher rates of comorbid anxiety and depression, all of which may increase the IBS severity [41]. Despite this, Black and Hispanic patients, who had markers of lower SES compared to the White and Asian patients in our study, were found to be underrepresented in this IBS cohort when compared with the hospital outpatient population at large.

We speculate that these findings could be in part caused by racial and financial disparities in healthcare access and utilization. A recent retrospective study in 2021 showed that minority (Black, Hispanic, and Asian) patients were less frequently referred for specialty visits for their IBS symptoms compared to White patients [42]. This is a well-known disparity that has been similarly reflected in minority patients with other GI conditions, including inflammatory bowel disease and colorectal cancer [43,44]. Minority patients and patients with lower SES also have lower levels of trust in the healthcare system and are generally less likely to seek medical care, potentially delaying or preventing diagnosis [45]. They have higher rates of hospitalization but fewer clinic visits, suggesting underutilization of ambulatory care for non-acute conditions such as IBS [46]. While this line of investigation was not the study objective, it warrants additional analysis of this racially diverse population at a safety-net hospital.

The most concrete barrier to care is insurance status itself: without proper coverage, patients of lower SES are unable to access and pay for consistent and secondary care. This is particularly relevant in the US, where access to care is dependent on the comprehensiveness of the insurance coverage [47]. Notably, data for our study were collected from 2005 to 2007, at which time a significant portion of BMC patients were uninsured. Universal healthcare coverage in Massachusetts was established within this time period in 2006/2007 and was not included in our IBS cohort. It would therefore be of great interest to assess the impact of the statewide insurance expansion on IBS epidemiology and demographics in this patient population after 2007.

Our study is the first to compare the distribution of IBS subtypes in a multiracial population. We found no significant racial differences in IBS subtype distribution. However, when compared with all non-Hispanic IBS patients, Hispanic patients had a significantly higher frequency of IBS-C. Other studies have also found differences in symptom characteristics of IBS across various racial and ethnic groups [25]. Some of these differences may be accounted for by racial and cultural variations in diet. For example, both Hispanic and Black Americans have been found to consume lower amounts of milk products and higher amounts of sodium than White Americans [48].

Dietary intake is a key component in IBS pathophysiology and is known to affect symptom severity and gut microbiome composition of IBS patients [49]. A recent study in 2022 found significantly elevated high-fructose corn syrup consumption among IBS patients in low SES communities, indicating that both cultural and financial dietary variations may impact IBS subtype distribution among different populations [50]. Additional investigation is needed to further elucidate the role of cultural factors and diet on IBS symptoms in the background of a multiracial and socioeconomically diverse population.

Even though this study is the first of its kind with several very important strengths and novel insights into IBS, there are some notable limitations, mainly related to the study design. Due to the retrospective nature of the study, IBS patients were identified by ICD diagnosis only, which may have resulted in the misclassification of patients with this condition. Additionally, the age of diagnosis was difficult to determine retrospectively and could contain inaccuracies. Accordingly, those results should be interpreted cautiously and future prospective study approaches could provide a remedy to this potential shortcoming. Finally, patients were classified into the four IBS subtypes based on their documented clinical presentation rather than by use of stool pattern questionnaires. Despite the excellent inter-investigator reliability when assessing the IBS subtype, misclassifications could have occurred based on a possible lack of data robustness.

In summary, our large, comprehensive study of IBS patients found significant differences in the prevalence rates and sociodemographic characteristics among the most prevalent four racial/ethnic groups. These may be attributable not only to innate cultural differences but also to systemic racial and socioeconomic disparities.

With growing awareness of the many disparities in healthcare, the effects of sociodemographic factors on long-term health outcomes have become increasingly apparent [51]. In DGBI disorders such as IBS, which are intimately associated with dysregulation of the brain–gut axis and psychosocial stressors, these effects have been linked to significant differences in symptom profiles, quality of life, and the degree of healthcare utilization [25,41,42,52,53]. As the first study to describe the epidemiology of IBS patients in a large academic safety hospital, our study contributes to IBS literature by identifying racial and socioeconomic patterns in IBS prevalence and thus highlighting systemic disparities that need to be addressed in the clinical management of IBS.

## Figures and Tables

**Table 1:** Patient Population at Boston Medical Center: Racial Distribution (%).

Race/Ethnicity	Outpatient Population at BMC 2007 *	IBS Sample **	*p* Value † <0.0001
White	32	70	
Black	34	14	
Asian	5	6	
Hispanic	17	10	

* Unique annual out-patients in 2007: N = 151,370; other ethnic groups were not included in this chart. ** Total IBS N = 740. Missing data for race in IBS sample N = 39; 1 Native Americans were not included in the IBS sample. † Chi-square test.

**Table 2:** Patient Population at Boston Medical Center: Racial Distribution (%) from 2005 to 2007.

Race/Ethnicity	Outpatient Population at BMC 2005 *	Outpatient Population at BMC 2006 **	Outpatient Population at BMC 2007 †	IBS Sample N = 740
White	33	32	32	70
Black	36	35	34	14
Asian	4	5	5	6
Hispanic	16	16	17	10

* Unique annual out-patients in 2005: N = 148, 086.** Unique annual out-patients in 2006: N = 151, 666. † Unique annual out-patients in 2007: N = 151, 370.

**Table 3:** Demographic Characteristics of IBS Patients at Boston Medical Center †.

Characteristics	All IBS (N = 740)	White (N = 492)	Black (N = 99)	Hispanic (N = 65)	Asian (N = 44)	*p* Value ††
Mean age (years [±SD)]	43 [±15]	41 [±16]	46 [±15]	46 [±15]	41 [±15]	0.003
Mean BMI [±SD] **	27 [±7]	27 [±7]	30 [±8]	27 [±5]	23 [±4]	<0.0001
Mean age at IBS dx (years [±SD] ‡	37 [±15]	36 [±14]	40 [±17]	42 [±15]	37 [±14]	0.06
Current age (%):						0.003
<30	27	30	17	12	23	
30–49	40	40	38	45	45	
50+	33	30	45	43	32	
Gender (%):						0.07
Male	25	23	24	34	36	
Female	75	77	76	66	64	
Marital status (%): *						0.009
Married	32	31	27	43	52	
Single	56	58	59	40	46	
Divorced	11	10	14	17	2	
Widowed	1	1	0	0	0	
Missing (N = 59)	–	–	–	–	–	
With children (%):*						0.03
Yes	38	35	50	51	37	
No	62	65	50	49	63	
Missing (N = 175 )	–	–	–	–	–	
Smoking Hx (%): *						0.14
Current	18	19	21	13	10	
Former	16	17	16	16	5	
Never	66	64	63	71	85	
Missing (N = 168)	–	–	–	–	–	
Insurance type (%): *						<0.0001
Private insurance	69	78	46	37	70	
Medicaid/Medicare	17	15	27	25	7	
None/Free Care	14	7	27	38	23	
Missing (N = 1)	–	–	–	–	–	
Highest level of education (%): *						<0.0001
Pre high school	2	1	4	10	0	
High school	7	2	26	40	5	
College	41	42	44	30	40	
Graduate/Professional	50	55	26	20	55	
Missing (N = 541)	–	–	–	–	–	
Employment (%): *						0.06
Unemployed	7	6	13	12	5	
Full time	29	27	30	38	41	
Part time	3	3	1	2	2	
Unspecified ‡‡	61	64	56	48	52	

† IBS sample comparison by race: missing data for race N = 39.One Native American was not included. * Missing data refer to the total IBS cohort of 740 patients only. ** Missing data for BMI for the total IBS cohort N = 213; for Whites N = 156; for Blacks N = 13; for Hispanics N = 14; and for Asians N = 16. BMI is kg body weight/body surface area in m^2^. ‡ Missing data about patient age at IBS dx for the total IBS cohort N = 289; for Whites N = 186; for Blacks N = 55; for Hispanics N = 22; and for Asians N = 13. †† Chi-square test. ‡‡ Unspecified employment also includes students.

**Table 4:** BMI Analysis of IBS Patients at Boston Medical Center (%) *.

BMI Category	All IBS N = 740	White N = 492	Black N = 99	Hispanic N = 65	Asian N = 44	*p* Value ** <0.0001
Underweight (<19)	5	5	1	6	11	
Normal (19–<25)	41	45	35	22	64	
Overweight (25–<30)	29	27	21	51	21	
Obese (≥30)	25	23	43	21	4	

* Missing data for BMI for the total IBS cohort N = 213; for Whites N = 156; for Blacks N = 13; for Hispanics N = 14; and for Asians N = 16. BMI is kg body weight/body surface area in m^2^. ** Chi-square test.

**Table 5:** IBS Subtype Distribution in Different Racial/Ethnic Groups (%) *.

Subtype **	All IBS N = 740	White N = 492	Black N = 99	Hispanic N = 65	Asian N = 44	*p* Value † 0.20
IBS-D	187 (25%)	132 (27%)	23 (23%)	11 (17%)	12 (27%)	
IBS-C	159 (22%)	97 (20%)	20 (20%)	21 (32%)	11 (25%)	
IBS-M	96 (13%)	56 (11%)	17 (17%)	11 (17%)	7 (16%)	
IBS-U	298 (40%)	207 (42%)	39 (40%)	22 (34%)	14 (32%)	

* IBS sample comparison by race: missing data for race N = 39; 1 Native American were not included. ** IBS-D = IBS with diarrhea, IBS-C = IBS with constipation, IBS-M = IBS with mixed bowel habits, IBS-U = unsubtyped IBS. IBS subtype was determined as described in the Section 2. † Chi-square test.

**Table 6:** IBS Subtype Distribution: Hispanics vs. Non-Hispanics (%).

Subtype	Hispanics N = 65	Non-Hispanics * N = 635	*p* Value † 0.02
IBS with constipation	21 (32%)	128 (20%)	
IBS with no constipation **	44 (68%)	507 (80%)	

* Non-Hispanics include Whites, Blacks, and Asians. ** IBS with no constipation includes IBS with diarrhea, IBS with mixed bowel habits, and unsubtyped IBS. † Chi-square test.

## Data Availability

The original contributions presented in the study are included in the article, further inquiries can be directed to the corresponding author/s.
